# Dangerous demographics in post-bleach corals reveal boom-bust versus protracted declines

**DOI:** 10.1038/s41598-021-98239-7

**Published:** 2021-09-22

**Authors:** Juliano Morais, Renato A. Morais, Sterling B. Tebbett, Morgan S. Pratchett, David R. Bellwood

**Affiliations:** 1 Research Hub for Coral Reef Ecosystem Functions, James Cook University, Townsville, QLD 4811 Australia; 2 College of Science and Engineering, James Cook University, Townsville, QLD 4811 Australia; 3 ARC Centre of Excellence for Coral Reef Studies, James Cook University, Townsville, QLD 4811 Australia

**Keywords:** Climate-change ecology, Population dynamics, Marine biology

## Abstract

Thermal-stress events have changed the structure, biodiversity, and functioning of coral reefs. But how these disturbances affect the dynamics of individual coral colonies remains unclear. By tracking the fate of 1069 individual *Acropora* and massive *Porites* coral colonies for up to 5 years, spanning three bleaching events, we reveal striking genus-level differences in their demographic response to bleaching (mortality, growth, and recruitment). Although *Acropora* colonies were locally extirpated, substantial local recruitment and fast growth revealed a marked capacity for apparent recovery. By contrast, almost all massive *Porites* colonies survived and the majority grew in area; yet no new colonies were detected over the 5 years. Our results highlight contrasting dynamics of boom-and-bust vs. protracted declines in two major coral groups. These dangerous demographics emphasise the need for caution when documenting the susceptibility and perceived resistance or recovery of corals to disturbances.

## Introduction

Climate change is rapidly transforming global ecosystems^1,2^. On coral reefs, bleaching-induced coral mortality has led to abrupt changes in their structure, biodiversity, productivity and functioning^3–7^. However, the majority of studies examining coral population dynamics have been based on coral cover or colony counts^1,6,8–11^. Only rarely is the fate of individual colonies considered over multiple years, especially during the critical post-bleaching ‘recovery’ period^12–15^. Long term evaluations of colony level changes enable the separation of immediate vs. delayed and partial vs. total colony mortality^16,17^. Furthermore, if considered across multiple bleaching events, colony-tracking may reveal cumulative impacts and allow the identification of genus and colony-level variation in the response to bleaching impacts.

**Figure 1:**
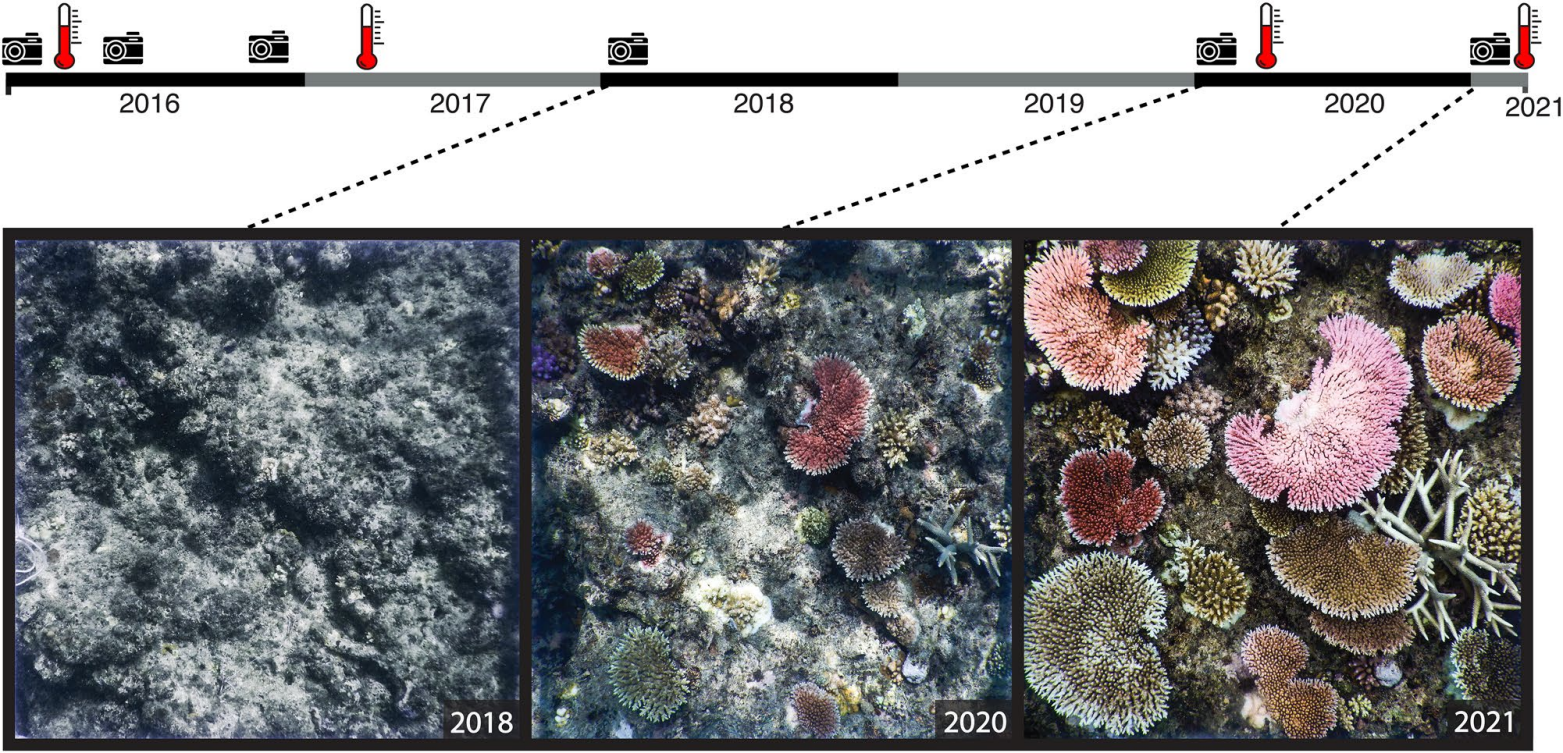
Timeline of the study with data collection instances (camera icons) and bleaching events (temperature gauges) with examples of quadrats (1 m^2^) of the same reef section across repeated sampling periods showing the growth of new *Acropora* colonies. January 2018, 24 months after first sampling, January 2020, 48 months after first sampling, and February 2021, 60 months after first sampling. All photographs taken at Lizard Island by SB Tebbett.

Using an extensive spatial design of fixed photo-quadrat locations (Fig. 1), we tracked the fate of 1069 coral colonies (in 362 quadrats spread across 16 km^2^ on the Lizard Island reef complex) over 5 years (2016–2021), encompassing three mass bleaching events on the Great Barrier Reef (GBR). Lizard Island was at the epicenter of the first of these bleaching events on the GBR, and represents a critical arena in which to explore long-term responses of corals to bleaching^1,18^. We focus on colonies within two dominant coral genera, with contrasting life-histories and differences in bleaching susceptibility: massive *Porites*, which are slow-growing^19^ and resistant to bleaching^20^, and *Acropora* (all growth forms)*,* which are fast growing but susceptible to bleaching^19,21,22^. Our goal was to evaluate the extent, magnitude and variability of colony-level susceptibility to successive bleaching events, as well as the potential demographic consequences and their implications for recovery.

## Results and discussion

**Figure 2:**
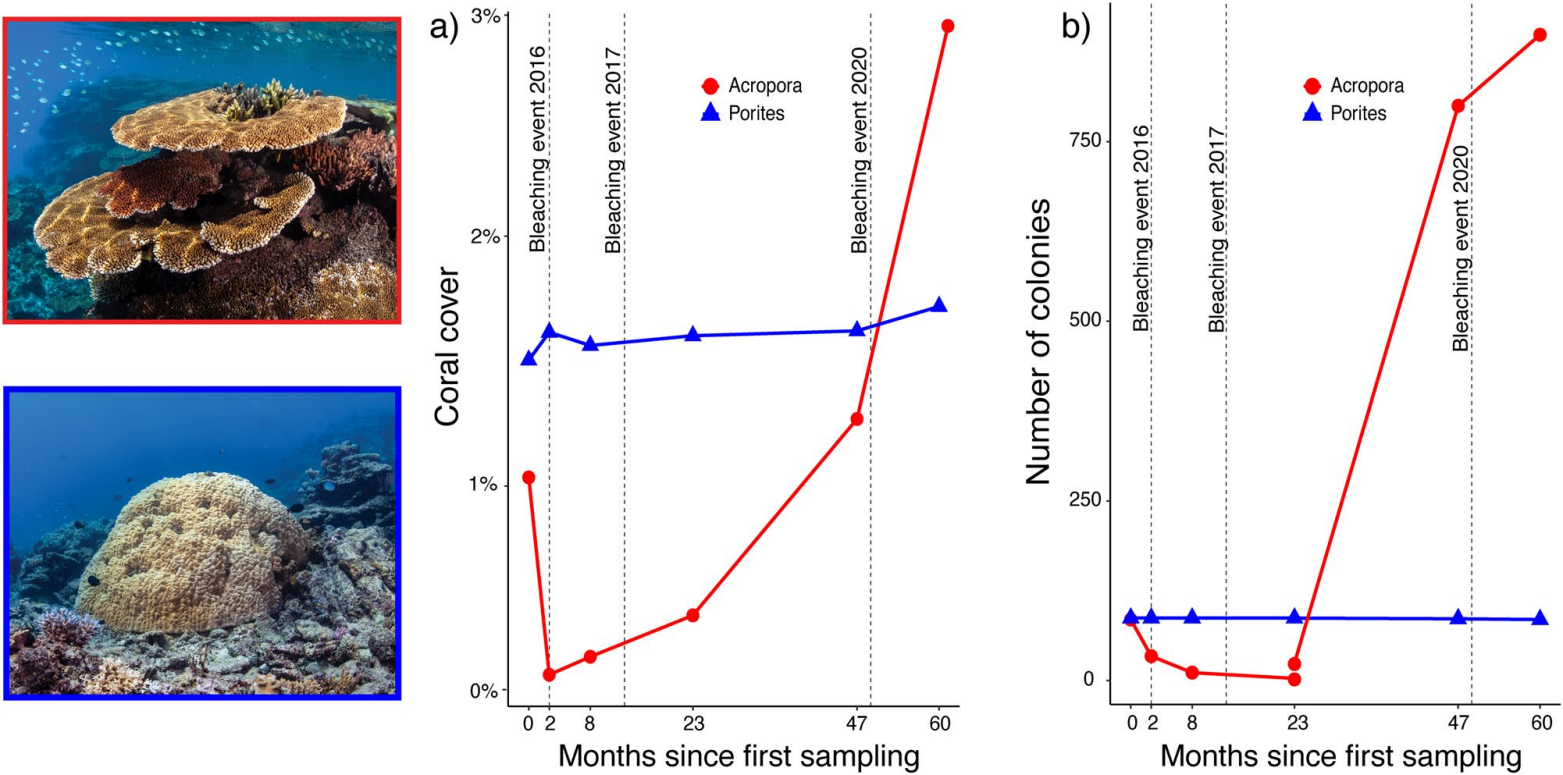
(**a**) Coral cover of *Acropora* and massive *Porites* based on 362 quadrats over the 60 month time period. (**b**) Total number of *Acropora* and massive *Porites* coral colonies tracked over the 60 month time period spanning three bleaching events at Lizard Island, northern Great Barrier Reef. Photographs: Victor Huertas.

**Figure 3:**
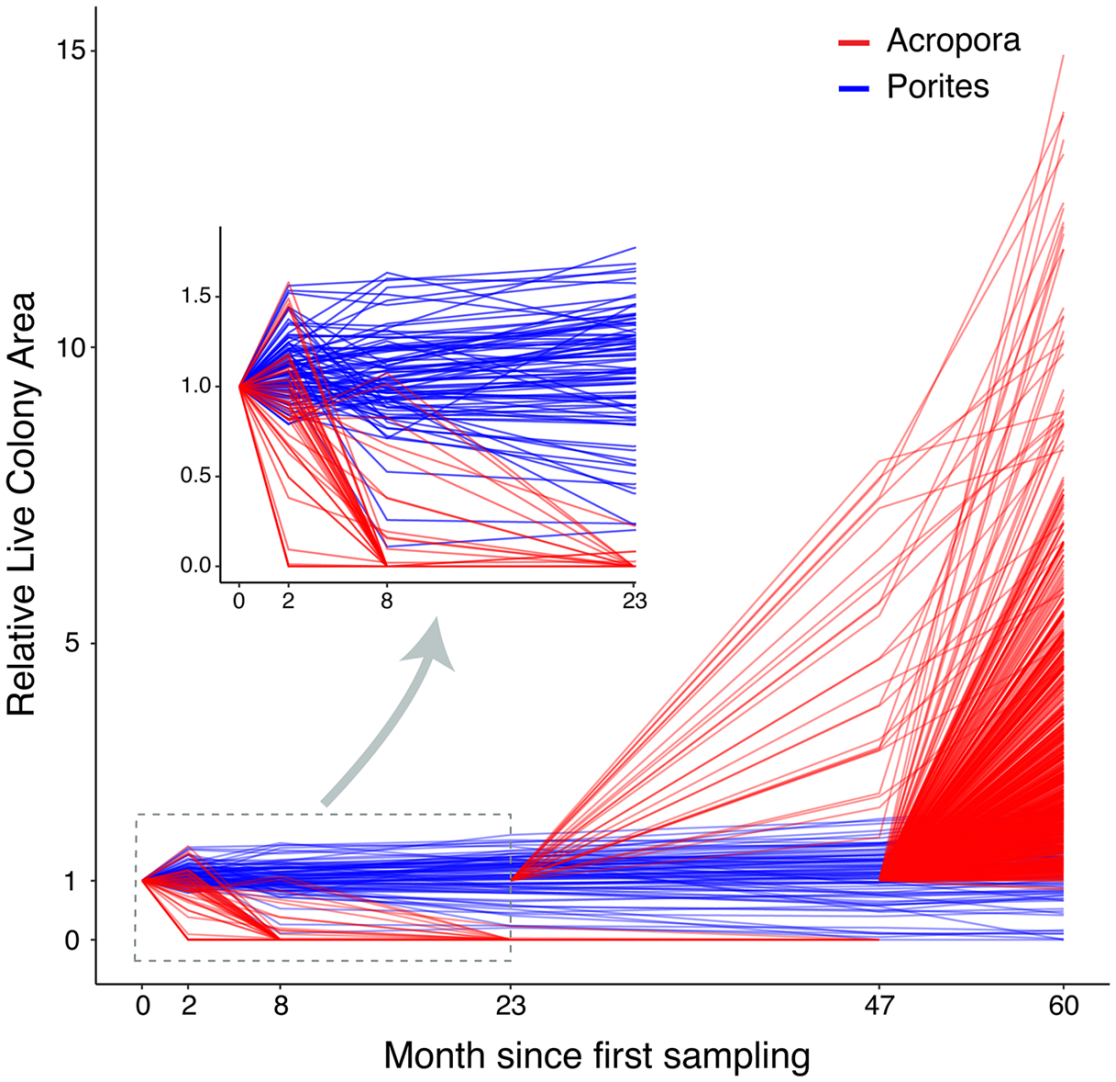
Relative live colony area of *Acropora* and massive *Porites* colonies over 60 months (each line represents an individual colony). Relative live colony area is the horizontal planar area of living tissue on a colony relative to the value at first detection. The small inner graph represents a zoom showing the standardized live area of *Acropora* and massive *Porites* colonies during the first 24 months since first sampling.

There were dramatic differences in the response to successive bleaching between the two coral types investigated (Fig. 2). *Acropora* colonies underwent complete local extirpation (i.e., 100% loss across all quadrats) in the 2 years following the first bleaching episode. Remarkably, however, there was also massive recruitment (i.e., the appearance of previously undetected colonies greater than 3 cm^2^) of *Acropora* starting 2 years after the first bleaching, resulting in a 1000% increase in the number of colonies relative to the start of the study (Fig. 2b). New colonies showed rapid growth, with an average 201% increase in colony size per year by the end of the study period (Fig. 3). Despite a tenfold increase in numbers and rapid growth, mean Acropora cover only increased from approximately 1% to 3%. Thus, it still remained low (< 3%) compared to historical levels of *Acropora* cover (from ~ 15 to 30% between 1995 and 2014^10^, likely reflecting an early ‘recovery’ trajectory (Fig. 2a).

**Figure 4:**
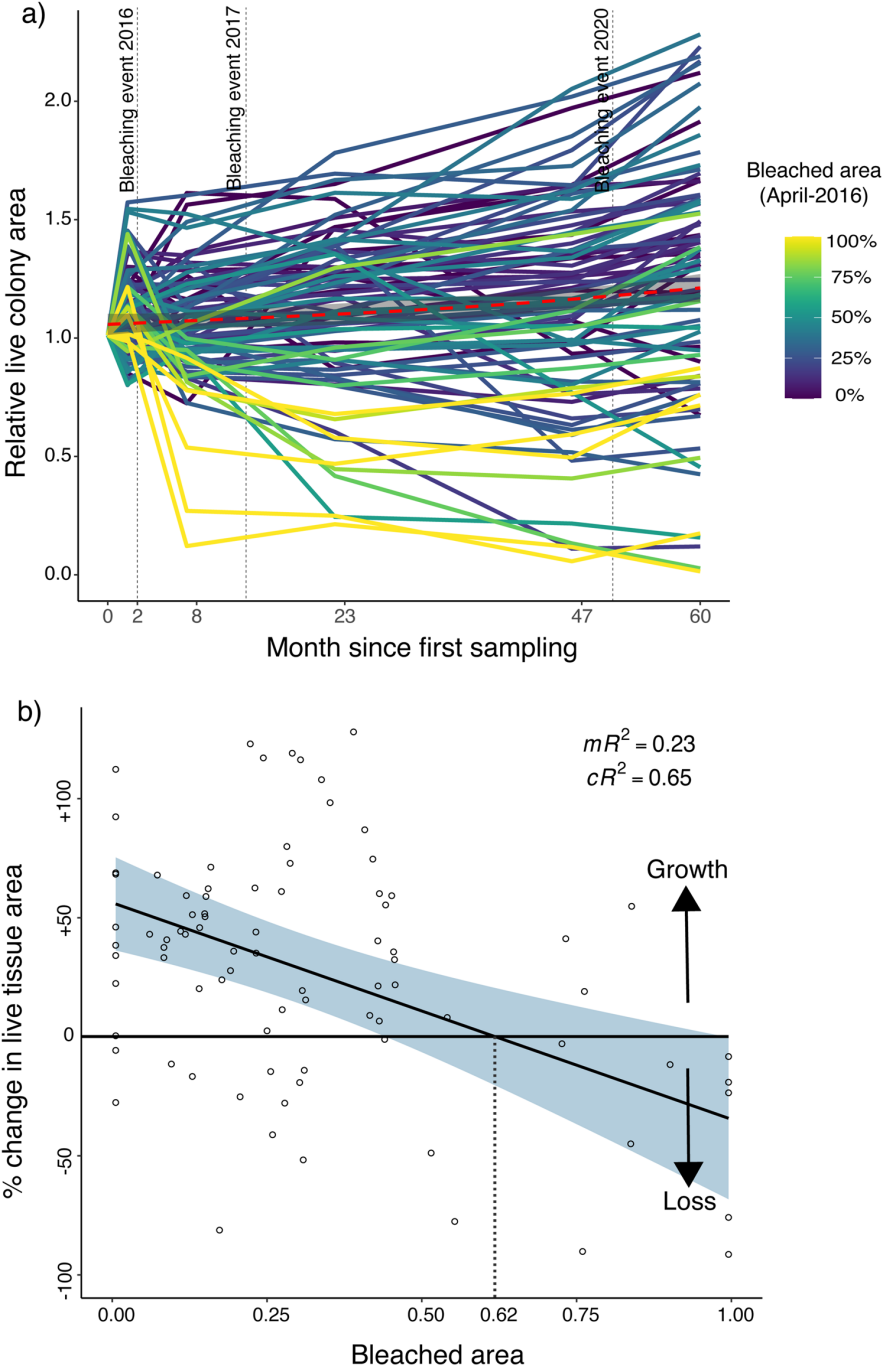
(**a**) Relative area of live tissue on massive *Porites* colonies over 60 months. Each line represents a single colony, with line colors representing the proportion of bleaching in each colony (during the 2016 bleaching event). The red dotted line represents the average increase of 21% in colony area of massive *Porites*. (**b**) Effect of the proportional bleached area (in April-2016) on the subsequent relative change in live tissue area of massive *Porites*. Line and band show the prediction and 95% confidence intervals of a Gamma GLMM, while dots show raw data points. Modelling was performed in the software R^34^, using the glmmTMB package^35^. The solid horizontal line and arrows indicate where colonies effectively increased or decreased live tissue area. The dotted vertical line represents the minimum bleached area required, on average, to trigger tissue loss. mR^2^ = marginal R^2^ and cR^2^ = conditional R^2^.

By contrast, the number of massive *Porites* colonies remained stable: there was only a 2.3% loss of colonies (2 colonies). But no new colonies were detected over the 5 years (Fig. 2). Surviving colonies showed an average increase in colony area of 21%, however, there was extensive among-colony variation in live tissue area changes (Fig. 3). Indeed, approximately half of the colonies suffered tissue loss. The extent of tissue loss was relatively well predicted by bleaching severity at the individual level (i.e., relative area of bleached tissue in the April 2016 bleaching event, Fig. 4). Thus, *Acropora* corals appear to be responding with a pronounced boom-and-bust pattern^23,24^, while massive *Porites* colonies exhibit a precarious degree of resilience, increasing in area but with an underlying recruitment deficit and a strong negative response in tissue area to bleaching severity (Fig. 4b).

Our findings agree with previous studies that show a high-susceptibility to thermal stress in *Acropora*^25–27^ and a degree of resistance to thermal stress in *Porites*^25,28^. These contrasting responses to disturbances play an important role in structuring coral communities and are now more apparent than ever given the frequency and severity of disturbances impacting coral reefs^24,29^. While the devastating effects of climate change on corals have been emphasized numerous times^1,6,10,26,30^, the fate of individual coral colonies has rarely been tracked over multiple bleaching events over multiple years, particularly in conjunction with key demographics traits such as recruitment and growth. Quantifying these dynamics is critical to understand future trajectories of coral populations subject to changing disturbance regimes, especially in a scenario of shortening ‘recovery’ windows^31–33^.

*Acropora* colony density at the start of the study was relatively low (85 trackable colonies, > 3 cm, across 362 quadrats (521.2 m^2^) in 2016). This was primarily due to two back-to-back cyclones in 2014 and 2015^10^. Following these disturbances, the severe bleaching events in 2016 and 2017 led to complete loss of *Acropora* in our censused area. After this widespread mortality period, we documented a > tenfold increase in colony numbers between 2018 and 2021 (relative to the first sampling period), with 897 new colonies by 2021 (1.72 new colonies m^−2^). These seemingly high levels of population replenishment were observed despite large (89%) declines in coral settlement across GBR, especially in *Acropora*, following the bleaching events in 2016 and 2017^32^. It was anticipated that the GBR-wide decline in settlement would have severely compromised the recovery capacity of these corals, as it was estimated that recovery would take at least a decade, even for faster-growing corals such as *Acropora*^32^. Although coral replenishment can be highly variable across spatial scales^32,36^, the rate of appearance of new colonies in our study, especially following such a sharp decline in coral numbers, offers some hope for the future of coral reefs.

Not only did new colonies of *Acropora* recruit in substantial numbers, but they also rapidly increased in size. Colonies initially detected in January 2018 had grown, on average, by 393% over 24 months. Peak detection of new colonies occurred in January 2020, and new colonies detected in 2020 and 2018 grew, on average, 211% between January 2020 and January 2021 (Fig. 3). Such fast growth is likely to underpin the perceived ‘potential recovery’ of *Acropora,* even as these ‘recovery’ windows between disturbances become shorter and shorter^32^. However, the realized long-term recovery of reef systems will depend on the capacity of these corals to persist in a scenario of increased frequency of extreme thermal events over the coming years^24,37^. The growth we observed resulted in a mean *Acropora* cover of just 3%, far below pre-bleach levels of coral cover. It may represent, therefore, just a short-term boom in a new Anthropocene configuration, where fast-growing corals persist but are unlikely to attain their former abundance due to successive disturbances and suppression of recovery dynamics^6,24,38^. Nevertheless, the responses we observed over five years highlight the remarkable potential for ‘boom and bust’ dynamics in *Acropora*, providing evidence that degraded coral reefs may still maintain some potential for apparent *Acropora* recovery, at least for a limited time and at the colony level.

However, our findings also highlight the need for caution. Although massive *Porites* shows ecosystem-level resistance to bleaching, responses of individual colonies are highly variable^24,25^. Indeed, individual bleaching susceptibility (indicated by the maximum proportion of colony area observed to bleach) was able to predict long-term (60 month) individual massive *Porites* colony tissue loss (Fig. 4a). Colonies that bleached more intensely also suffered heavier tissue loss, while those that bleached less intensely often grew in tissue area (Fig. 4b). Nevertheless, even when massive *Porites* colonies suffered intermediate to high bleaching (in proportion to live colony area), their likelihood of recovery was much higher than *Acropora* colonies as noted previously^25^. Most importantly, however, despite censusing 521.2 m^2^ of reef in extreme detail over 5 years, we did not record a single new massive *Porites* colony. This lack of apparent recruitment over half a decade suggests that massive *Porites* could be rare, a pattern supported by the examination of coral recruitment on tiles across large spatial scales post-bleaching^18^. However, the apparent rarity of *Porites* recruits could also be magnified by the difficulty of detecting *Porites* recruits in photos. Indeed, due to a combination of cryptic colouration, small size and slow growth, *Porites* recruits are likely to be harder to detect than *Acropora* recruits in photographs, potentially leading to an underestimation of relative recruitment in *Porites*^32,39^. Nevertheless, the scarcity of massive *Porites* recruitment throughout our study highlights the potential for protracted declines and storage effects^40,41^. Such protracted declines may be even more concerning than sudden dynamic shifts, as in *Acropora* abundance, as they may be easier to overlook or ignore, and harder to reverse^42^.

Thus, our data has revealed how the colony-level population dynamics of two archetypical coral types, massive *Porites* and *Acropora,* have responded in distinctly different manners over multiple disturbances events caused by thermal stress and a short-term ‘recovery’ window. For weedy, fast-growing *Acropora* colonies, high susceptibility to bleaching and complete mortality was followed by substantial recruitment and fast growth, revealing a marked capacity for apparent ‘recovery’. However, the lifespan of these new colonies is already being tested as a fourth bleaching event began to unfold in January/February 2021, with marked paling of these new *Acropora* colonies (Supplementary Fig. 1). We also demonstrated the well-documented resistance of stress-tolerant colonies of massive *Porites*, with net positive growth over five years. However, the complete lack of new colonies over this same time frame (despite intensive sampling) suggests that recruitment is rare and, potentially, unpredictable. Without replacement, increasing repetitive bleaching events^30,43^, may drive a slow, protracted decline of massive *Porites* that could be easily overlooked. These markedly different demographic patterns offer grounds for both optimism and concern. Massive *Porites* are resistant, but potentially compromised in the long-term, while *Acropora* are vulnerable, but have greater capacity to recover in the aftermath of major disturbances^24,31^. In both cases their dangerous demographics require caution when interpreting the susceptibility and perceived resistance of corals to disturbances.

## Methods

### Study area and sampling

Tracking of individual colonies was based on a comprehensive photo-quadrat census at Lizard Island, (14°40′ S, 145°28′E) in the northern region of the Great Barrier Reef (GBR), described in Wismer et al.^44,45^. This region experienced two prolonged thermal events between February and April 2016, as well as between January and March 2017, leading to extensive coral bleaching^1,10^. During the first sampling period, a total of 19 permanent transects (between 50 and 210 m in length, as constrained by reef morphology) along the reef ‘crest’ (at 0–4 m below chart-datum) were established around Lizard Island (Supplementary Fig. 2). Along each transect, between 12 and 38 quadrats (1 m^2^ area), approximately 5 m apart, were sampled. These transects were revisited five times: in April 2016 (2–3 months after first sampling); October 2016 (9 months after first sampling); January 2018 (after 24 months); January 2020 (after 48 months) and January/February 2021 (after 60 months).

Using SCUBA, each quadrat was photographed in each of the six sampling periods (Camera: Nikon Coolpix AW130) from a planar ‘bird's-eye’ view between 09:00 and 16:00 h. To survey each transect on subsequent trips, the starting location was identified based on a GPS mark taken on the first sampling trip. The same quadrat area was relocated during each sampling trip using a second underwater camera containing all previous images from each quadrat ordered from the start to the end of the transect (see Wismer et al.^44^ for a sensitivity analysis of this method). A total of 362 photo-quadrats were sampled across the entire study. Around each quadrat there was also a buffer area (Fig. 1) where individual colonies could be located and followed. We therefore tracked and quantified the fate of individual colonies within the quadrats and in the 10 cm wide buffer area around each quadrat. This resulted in a censused area of 1.44 m^2^ per quadrat, and a total censused area of 521.2 m^2^. All colonies from the two studied groups (*Acropora* spp. and massive *Porites* spp.) within the photo-quadrats and within the buffering area were recorded, identified (to species level whenever possible) and had their live tissue area determined from the photographs (please see Supplementary Fig. 3). Live tissue area of each colony was determined by tracing around the visible live coral tissue to obtain the planar area in cm^2^ relative to the quadrat area (10,000 cm^2^). All images were processed using the software ImageJ^46^.

### Data analysis

We considered ‘growth’ to be the difference in live tissue area of each colony between the first and subsequent sampling periods. For *Acropora* colonies, growth was calculated from recruitment onwards (for colonies that recruited in 2018 or 2020), and was expressed as increase in live tissue are per year. Because there were no recruits detected for *Porites*, all colonies were present at the start of the study. Thus, growth for massive *Porites* colonies represents growth over 5 years. To facilitate comparisons among colonies, we standardized live tissue area using the area from the first sampling period as the reference. The same method was used when we detected ‘recruit’ colonies (i.e., new colonies of a visible size in the quadrat) in the subsequent samples.

For massive *Porites* colonies that bleached, we also measured the proportion of bleached planar area on each colony during the 2016 bleaching event. We tested for the hypothesis that bleaching severity had an effect on the change in live tissue area (loss or gain) for massive *Porites* colonies following this event. ‘Bleaching severity’ was defined as the highest proportion of tissue area observed to bleach for each coral colony across all trips in which bleaching occurred. To test this hypothesis, we used a generalized linear mixed effects model (GLMM) with tissue area change as the response variable and ‘bleaching severity’ as the predictor. We also included quadrat, nested in transect, as random intercepts in the model to account for repeated sampling and any lack of spatial independence in the data. We used a Gamma error distribution with a log link function. Because the data was slightly right skewed, we used model selection to find the best error distribution to fit the model. We compared models fitted using the Gaussian distribution, the lognormal distribution (i.e., a Gaussian distribution with a log link), and the gamma distribution (also with a log link), using Akaike’s Information Criterion. Model selection showed that the gamma distribution model was the one that best balanced fit and parsimony, and therefore was the one chosen. Model fit and assumptions were assessed using residual plots, all of which were satisfactory. Statistical modelling was performed in the software R^34^, using the glmmTMB package^35^.

## Supplementary Information

Supplementary Information.
