# Supplementary material for: Dangerous demographics in post-bleach corals reveal boom-bust versus protracted declines
Source: Sci Rep. 2021 Sep 22;11:18787. doi: 10.1038/s41598-021-98239-7 (PMC8458526; doi:10.1038/s41598-021-98239-7)
Supplement: Supplementary file 1 — Supplementary Information. [file 41598_2021_98239_MOESM1_ESM.docx]

Supplementary Information

**Dangerous demographics in post-bleach corals reveal boom-bust versus protracted declines**

Juliano Morais^1,2,3^, Renato A. Morais^1,2,3^, Sterling B. Tebbett^1,2,3^, Morgan S. Pratchett^3^, David R. Bellwood^1,2,3*^

^1^ Research Hub for Coral Reef Ecosystem Functions, James Cook University, Townsville, Queensland 4811, Australia

^2^ College of Science and Engineering, James Cook University, Townsville, QLD 4811, Australia

^3^ ARC Centre of Excellence for Coral Reef Studies, James Cook University, Townsville, QLD 4811, Australia


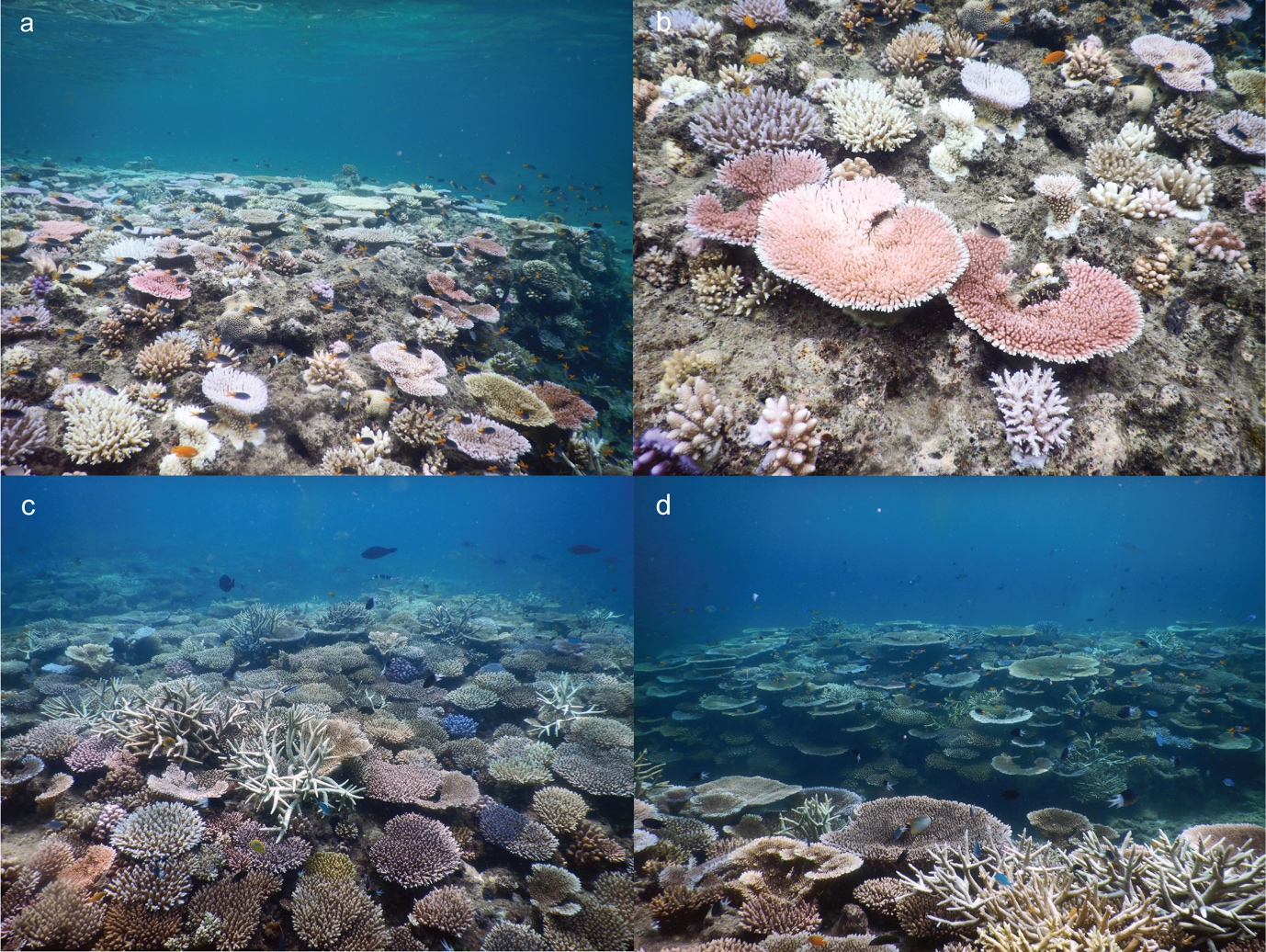


Supplementary figure 1**. Clear signs of bleaching in *Acropora* colonies at Lizard Island (a, b).** Both photos show signs of mild bleaching in *Acropora* colonies in February 2021. **And an example of a potential source reef of coral planulae, Linnet Reef, approximately 15 km from Lizard Island (c, d).** Note the larger colonies on Linnet Reef, photographed in January 2021, which may have survived the 2016 bleaching event. All photographs SB Tebbett.

Supplementary figure 2. **Map of study sites**. Map of Lizard Island, located in the northern region of GBR, showing the 19 transects in our study. Sites are represented by red circles and indicate the location of an individual transect.


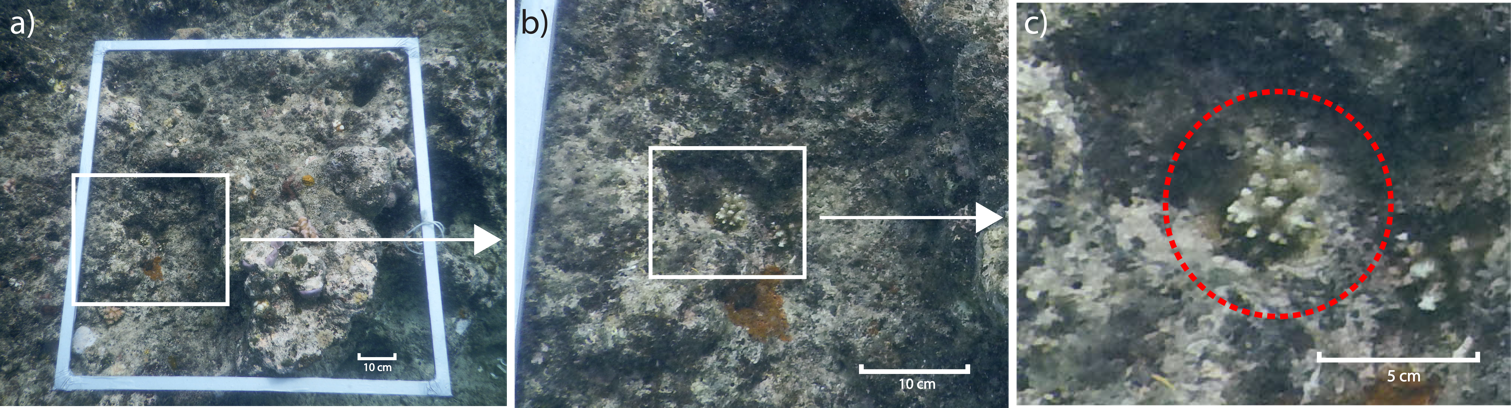


Supplementary figure 3. **Examples of images used to count and measure recruits of *Acropora* colonies**. The sequence of images shows increasing levels of magnification of the same photograph, with close-up views of an individual coral colony recruit. All photographs taken at Lizard Island by SB Tebbett.

Supplementary table 1. **List of likely *Acropora* species found in our sampling area**. This table shows the likely *Acropora* species occurring in our sampling area based on morphological characteristics and using Lizard Island field guide (<http://lifg.australianmuseum.net.au/Hierarchy.html>) for identification.

| **Species**  *Acropora carduus*  *Acropora cerealis*  *Acropora cytherea*  *Acropora divaricata*  *Acropora elseyi*  *Acropora florida*  *Acropora gemmifera*  *Acropora grandis*  *Acropora humilis*  *Acropora hyacinthus*  *Acropora intermedia*  *Acropora latistella*  *Acropora loripes*  *Acropora millepora*  *Acropora monticulosa*  *Acropora muricata*  *Acropora nasuta*  *Acropora samoensis*  *Acropora sarmentosa*  *Acropora selago*  *Acropora spathulata*  *Acropora tenuis*  *Acropora valenciennesi*  *Acropora valida*  *Acropora willisae*  *Acropora yongei* |
| --- |

Supplementary text 1. **Likely species of massive *Porites* in our sampling area.** For massive *Porites*, the likely species occurring in our sampling area were *Porites australiensis, Porites lobata* and *Porites lutea*. However, it is difficult to tell these three species apart with massive *Porites* being one of the most difficult coral groups to separate without molecular analysis. These identifications are also based on the Lizard Island field guide.
